# Cyanoacrylate Glue as an Alternative for Peritoneal Closure

**DOI:** 10.7759/cureus.71893

**Published:** 2024-10-19

**Authors:** Oscar A Ledesma-Orta, Mauricio L Rendon-Saldivar, Mauricio A Saldaña-Ruiz, Alejandro Garza-Alvarez, Gerardo E Muñoz-Maldonado

**Affiliations:** 1 General Surgery, Dr. José Eleuterio Gonzalez University Hospital at the Autonomous University of Nuevo León, Monterrey, MEX; 2 Medicine, Dr. José Eleuterio Gonzalez University Hospital at the Autonomous University of Nuevo León, Monterrey, MEX; 3 Gastroenterology, Dr. José Eleuterio Gonzalez University Hospital at the Autonomous University of Nuevo León, Monterrey, MEX

**Keywords:** cyanoacrylates, general surgery, hernia, herniorrhaphy, surgical mesh

## Abstract

Inguinal hernia repair is one of the most common surgical procedures. Traditionally, traumatic fixations have been used to prevent mesh migration, resulting in chronic pain in the groin. However, a non-traumatic alternative with cyanoacrylate glue is offered.

A 50-year-old male patient underwent elective surgery for inguinal hernia repair. During the surgery, it was decided to use cyanoacrylate glue for mesh fixation without complications. In his follow-up consultation, the patient showed a satisfactory course.

The use of cyanoacrylate glue, a synthetic resorbable adhesive, for more rapid wound closure has shown favorable results. It is characterized by a low rate of complications and a significant decrease in groin pain in the short- and medium-term. This technique is considered a viable and effective alternative for the reduction of complications, with promising clinical results.

## Introduction

Inguinal hernia repair is one of the most performed surgical procedures in the world. The potential benefits of the laparoscopic approach with early postoperative recovery and decreased incidence of long-term groin pain are well known [1]. To prevent mesh migration and therefore recurrence, there are traumatic fixation methods that may be associated with chronic groin pain and non-traumatic fixation methods such as fixation with fibrin and cyanoacrylate glues [2].

The first report of the use of cyanoacrylate for mesh fixation in laparoscopic inguinal hernioplasty dates to 1998 with an initial experience of seven patients [3]. Currently, its use and indication have been extended, enabling an evolution in the characteristics and methods of transoperative application [4]. Due to its high tensile strength, which favors the adhesion of solid tissue, as well as its impermeability to blood and other liquids, it is a safe option to consider in this type of procedure. We report a case of laparoscopic inguinal hernia repair in which we use cyanoacrylate glue for mesh fixation.

## Case presentation

**Figure 1:**
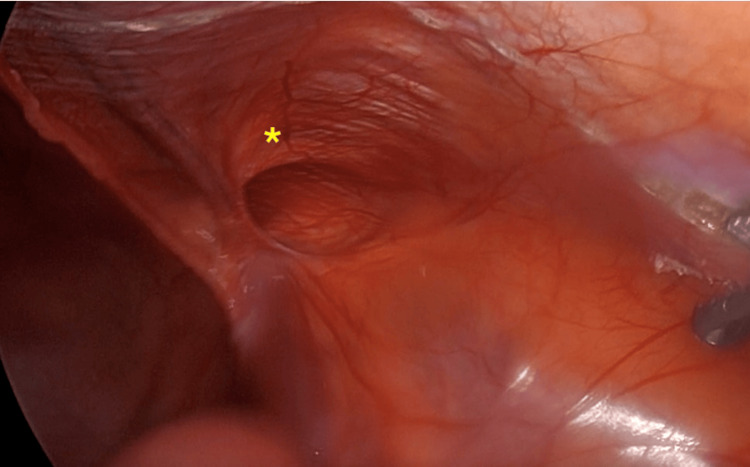
Diagnostic laparotomy showing lateral defect to epigastric vessels corresponding to indirect inguinal hernia (yellow asterisk)

**Figure 2:**
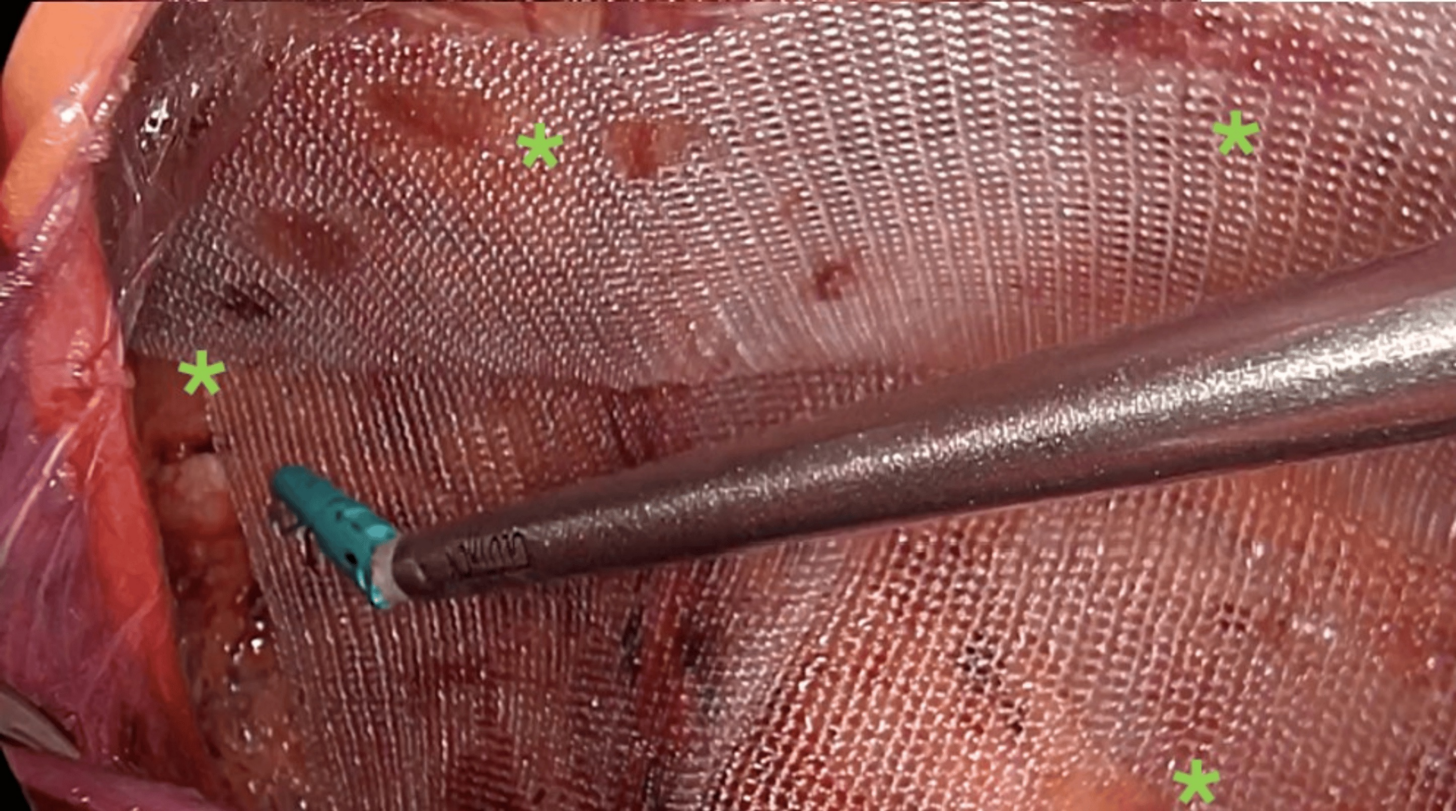
Fixation of the mesh using cyanoacrylate glue (green asterisks)

**Figure 3:**
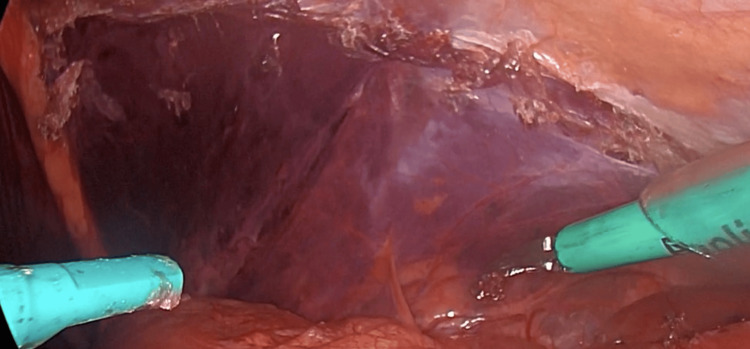
Complete visualization of the peritoneum at the end of the closure with cyanoacrylate

A 50-year-old male patient with no relevant medical history was admitted for elective outpatient surgery for inguinal hernia mesh repair. Per the established surgical protocol of asepsis and antisepsis, the surgery was performed under general anesthesia, starting with the pneumoperitoneum through a Veress needle of 15 mmHg in the umbilical region. Then, an 11 mm trocar was introduced in the umbilical region followed by two 5 mm trocars at the level of the left and right midclavicular lines at the umbilical level. A diagnostic laparoscopy was performed to identify the hernia (Figure 1). We began the creation of the peritoneal flap 4 cm to 5 cm above the deep inguinal ring, continuing the dissection through zone 2, 1, and finally zone 3 (5). We reduced the content of the hernia and would have done the same if a lipoma of the cord existed; however, a lipoma was not found. Subsequently, vas deferens and spermatic vessels with adequate parietalization of the elements were identified. Having a critical view of the myopectineal orifice, a medium-weight polypropylene mesh of 12 cm x 15 cm was introduced and fixed with Glubran-2® (n-butyl-2-cyanoacrylate + methacrylosisolfolane; GEM LLC, Viareggio, LU, ITA) in a drip device (Glutack®; Gem LLC) with a five-second wait after each application (two drops per quadrant) to allow adequate polymerization (Figure 2). Then, the pneumoperitoneum was reduced to 8 mmHg for the closure of the peritoneum without tension, achieving an adequate approximation. The pneumoperitoneum was resolved under direct vision by observing the structure of the mesh and verifying adequate position and absence of folds (Figure 3).

The post-surgical physical examination was normal, with the presence of minor wounds secondary to the surgical procedure. The patient was discharged after adequate clinical evolution and was prescribed cephalexin 500 mg tablet orally every 12 hours for five days, two tablets of acetaminophen 500 mg tablet orally every eight hours for five days, tramadol 50 mg tablet every eight hours for three days, and follow-up with the general surgery consultation a week later. The following week, the patient returned for the follow-up and showed favorable evolution and no evidence of complications. Six months later, the patient is stable, with adequate wound healing and evolution.

## Discussion

For the closure of the peritoneum, materials such as absorbable sutures have historically been used, which implies the ability to perform intracorporeal suturing [5], the use of titanium clips [6], and absorbable tacks [7]. In this case, an alternative for peritoneal closure was proposed based on the safety demonstrated by the use of cyanoacrylate since 1998 [3,7] for atraumatic fixation of the mesh in the myopectineal orifice, regardless of a transperitoneal [3,7,8-10] or preperitoneal [11] approach.

There are limited publications in the medical literature regarding the closure of the peritoneum with the use of glues composed of fibrin and/or cyanoacrylate [2-12], obtaining favorable results by achieving closure of the peritoneum with a low rate of complications, secondary to the fact that it is a non-traumatic procedure. In this case, we observed less postoperative pain and a reduction in the time required to achieve adequate closure by not performing intracorporeal suturing. Furthermore, chronic pain was less frequent at the third-month follow-up, which is in accordance with the meta-analysis performed by Antoniou et al. [2], which showed less groin pain when cyanoacrylate was used. The clinical study performed by Sanchez-Grau et al. [13] demonstrates that the use of cyanoacrylate is safe for closing the peritoneum with less postoperative pain in the short- and medium-term. It has also been documented that allergic reactions to topical cyanoacrylate are rare, but if they do occur, treatment may be difficult, for which oral steroids are used [14]. Our case shows evidence of a feasible option to facilitate the closure of the peritoneum after transabdominal preperitoneal (TAPP) hernioplasty, which was achieved in nine cases with complete closure without the need for another additional method, the absence of complications at the third-month follow-up, and a low rate of groin pain as demonstrated in the international literature [2].

Cyanoacrylate is a synthetic sealant that polymerizes quickly and is resistant when in contact with organic or liquid tissues. Its total degradation occurs through hydrolysis, and the duration varies according to the type of tissue and the amount applied. This glue is suitable because it does not interfere with tissue healing and causes a physiological inflammatory reaction in nearby tissues, making it better tolerated than sutures or staples, thereby reducing chronic pain after surgery [11].

## Conclusions

The use of cyanoacrylate for peritoneal closure is proving to be a viable and safe alternative, with the potential to become standard practice in minimally invasive surgeries. Its capacity to reduce surgical time while maintaining safety and efficacy makes it an attractive option for improving efficiency in the operating room. The absence of long-term complications further substantiates its viability, providing reassurance regarding its efficacy and safety. Nevertheless, while the initial results are promising, more research is needed to confirm its efficacy across a wider range of procedures and patient populations. Future studies should focus on comparing long-term outcomes in various types of surgeries and assessing any potential limitations.
